# Patient and Provider Perspectives on Barriers to Accessing Gynecologic Oncologists for Ovarian Cancer Surgical Care

**DOI:** 10.1089/whr.2020.0090

**Published:** 2020-12-28

**Authors:** Kristin Weeks, Michele West, Charles Lynch, Lisa Hunter, Chelsea Keenan, Savannah Borman, Megan McDonald, Mary Charlton

**Affiliations:** ^1^ Department of Epidemiology, University of Iowa College of Public Health, Iowa City, Iowa, USA.; ^2^ Carver College of Medicine, University of Iowa, Iowa City, Iowa, USA.; ^3^ Iowa Cancer Registry, University of Iowa, Iowa City, Iowa, USA.; ^4^ Department of Obstetrics and Gynecology, Division of Gynecologic Oncology, University of Iowa Hospitals and Clinics, Iowa City, Iowa, USA.

**Keywords:** gynecologic oncologist, ovarian cancer, surgical care, referral, rural, disparity

## Abstract

**Objective:**

National Comprehensive Cancer Network (NCCN) guidelines recommend that patients with ovarian cancer receive surgical care from a gynecologic oncologist. However, 15%–30% of patients with ovarian cancer do not receive surgical care from this specialist. The reasons for this remain unknown. We aim at assessing the barriers and attitudes perceived by patients with ovarian cancer who did not receive their primary surgery from a gynecologic oncologist and by diagnosing providers in an exploratory qualitative study.

**Materials and Methods:**

Patients and providers were sampled through the Iowa Cancer Registry. Participants were interviewed by telephone about barriers that patients face receiving surgical care from a specialist. Interviews were transcribed verbatim, and thematic analysis was completed by two team members.

**Findings:**

Providers (*n* = 10, 13% participation rate) identified many system-level barriers, including poor provider-to-provider communication, long time-to-surgery wait times, and a limited number of gynecologic oncologists working in their referral range. Patients (*n* = 16, 38% participation rate) denied system-level barriers; however, no patients reported receiving a referral to a gynecologic oncologist. This, in and of itself, constitutes a system-level barrier. Providers identified many barriers that their patients face, whereas patients failed to identify these barriers and denied facing them. Patients described the shock that they experienced after diagnosis and its limitations on their decision-making process. Both providers and patients agreed that the providers were influential in determining care decisions.

**Discussion:**

There is a divergence in the perceptions of barriers to care between providers and patients. Open discussions are needed about options and clinical guidelines for surgical ovarian cancer care. Further research is needed to develop and evaluate mechanisms to improve provider-to-patient discussions about surgical recommendations.

## Introduction

Ovarian cancer is the fifth leading cause of cancer death in women in the United States.^1^ One in 77 women in the United States will be diagnosed with ovarian cancer in their lifetime, and less than half will survive 5 years after diagnosis.^1^ Surgical treatment in accordance with clinical guidelines is an important prognostic factor for women with ovarian cancer.^2,3^

Gynecologic oncologists are physicians who were residency-trained in obstetrics and gynecology and fellowship-trained in gynecologic oncology to perform cancer surgery on patients with ovarian cancer.^3–8^ Specialty surgical care by a gynecologic oncologist has been associated with improved patient survival and greater adherence to clinical guideline recommendations.^3–8^ Current National Comprehensive Cancer Network (NCCN) clinical guidelines and other national organizations recommend that all women with stage IB-IV ovarian cancer receive surgical care from a gynecologic oncologist.^3,9–13^

Despite the evidence of improved outcomes and NCCN recommendations, as many as 30% of women with ovarian cancer in the United States and nearly 15% of women in Iowa are not receiving surgical care from a gynecologic oncologist.^11,14–20^ Researchers have hypothesized this discrepancy between recommended and received care is due to the incidental diagnosis, discovery of cancer during non-cancer surgery and disagreement about standards of care.^21,22^ Low referral rates may also be associated with receipt of care at a rural hospital or a low-volume hospital, and lower patient income or a lack of insurance.^3,7,22,23^ Finally, others have hypothesized that geographic disparities and distance impact access to care from a gynecologic oncologist.^11^

Assessing patient and provider barriers and attitudes in receiving care from a gynecologic oncologist may help develop evidence-based interventions to increase adherence to guideline-recommended surgical care within the state of Iowa. We aimed at assessing barriers and attitudes regarding referral to specialty care from both the patient and provider perspective. We then compared the perspectives of these two groups to identify areas where the perspectives were in conflict.

## Materials and Methods

We constructed purposive samples of providers and patients by using data from the Iowa Cancer Registry (ICR) until saturation was achieved. The ICR is a statewide cancer registry that has been part of the high-quality National Cancer Institute's Surveillance, Epidemiology, and End Results (SEER) Program since 1973. The ICR collects data on all SEER-reportable cancers diagnosed among Iowa residents.

We first identified five Iowa hospitals in areas with no practicing gynecologic oncologists where the highest numbers of ovarian cancer surgeries were performed from 2010 to 2016. We then identified an additional five Iowa hospitals without a gynecologic oncologist where low rates of ovarian cancer surgery were performed that best matched the former group on residency program status, bed size, and location in the state.

Eligible providers included hospital administrators, obstetric-gynecologists, and medical oncologists because we wanted to gather a variety of perspectives on issues related to referrals to gynecologic oncologists. Each was recruited in equal number; one of each provider type was sampled and recruited from each hospital. Second, we also identified providers who recently referred ovarian cancer surgical cases to the multi-specialist tertiary care center with a gynecologic oncologist division in our state; cases included both patients that had been and had not been previously operated on by non-gynecologic oncologist surgeons. Providers were sampled in sets of 10, including obstetric-gynecologists and medical oncologists, until saturation was achieved.

Rurality of practice is presented and defined by rural-urban commuting area codes from the 2010 decennial census and 2006–2010 American Community Survey.

Our patient sampling frame was constructed from residents of the state diagnosed with ovarian cancer in 2012–2016 who received their first ovarian cancer surgery at a hospital without a gynecologic oncologist surgeon and were still alive as of October 2019 according to ICR follow-up records. All potential patient participants were recruited.

Eligible subjects were mailed a letter containing information about the study and about our upcoming recruitment call. They were called by telephone 4–8 days after receiving the letter and asked to participate in the study. Telephone interviews with patients were conducted by one staff member at the ICR, and telephone interviews with providers were conducted by two members of the research team. All interviewers received training in conducting qualitative interviews. If the eligible subject agreed to participate, an interview was conducted during the initial call or at a time that was convenient within the following 2 weeks. Eligible participants were recruited with a total of four telephone calls to their preferred home telephone or cellphone number.

All subjects provided verbal consent to participate in the study and to have their interview audio recorded. This study was approved by the University of Iowa Institutional Review Board.

Interview questions were informed by a literature review of relevant studies and guided by the Theory of Reasoned Action and the Theory of Planned Behavior.^11,24–30^ While the Theory of Reasoned Action focuses on understanding attitudes, intentions, and beliefs as determinants of behaviors, the Theory of Planned Behavior accounts for factors beyond the interviewee's control that limit or facilitate one's behaviors and choices.

Our interview guides assessed system-level and patient-level barriers and facilitators of referrals to gynecologic oncologists, as well as factors considered during the surgeon selection decision-making process. We assessed the interviewee's attitudes and beliefs, as well as their perception of the attitudes and beliefs of the other parties involved in the decision-making process. Our questions were structured to focus on attitudes toward referral to or selection of a gynecologic oncologist.

Finally, given the Theory of Planned Behavior's hypothesis that perceived control of behaviors and choices at the time of decision-making can greatly influence actions, we assessed patient and provider perceptions of decision-making control and the level of influence the other party had in their decision-making process.

The provider interview guides were developed first; separate guides were developed for practicing obstetric-gynecologist physicians, medical oncologist physicians, and administrators (Supplementary Tables S1–S3). They included 12–14 open-ended interview questions with follow-up probes and one to two close-ended questions. Questions focused on understanding the referral decision-making process and the barriers to referring patients to gynecologic oncologists for surgical care. Interviews lasted an average of 20 minutes.

The patient interview guide (Supplementary Table S4) was developed by using a similar approach to the provider interview guides. However, based on the findings from the provider interviews that were conducted first, probes were developed to specifically ask about patient-level barriers that providers reported. This process allowed us to elicit patient reactions to each of the barriers that providers suggested patients experienced. Subject matter experts reviewed and pilot-tested all interview guides. Interviews were recorded and transcribed verbatim.

A thematic analysis approach was utilized to analyze the transcripts.^31,32^ Two research team members individually developed their own initial codes and selected their own representative quotations. The codes were compared by using grounded theory, the constant comparative method, and multiple coding, which involved each team member bringing their own observations and descriptions about the data to the team meeting and discussing their own codes in detail.^31,33,34^ Any differences in themes and ideas were discussed until consensus was reached. The provider and the patient themes were analyzed separately and then compared in a final step by multiple coders.

Based on literature for qualitative research sample size, we aimed at conducting at least 10–15 interviews for each group (providers and patients) until saturation was achieved.^35^ We analyzed interviews for each group in small batches to determine whether saturation had been reached.^35^ We did not find additional themes in the last batches analyzed, and consequently did not conduct further interviews for this exploratory qualitative study.^35^

## Results

Of the 75 providers who were sent recruitment letters, 10 participated in interviews, including five obstetrics and gynecology-trained physicians whose primary role was patient care, two obstetric and gynecologist physicians whose primary role was administration, one medical oncologist, and two non-physician administrators. Providers had been at their current positions for 5–33 years. Most practiced in urban locations and worked in practices affiliated with a hospital system (Table 1). Providers estimated that between one and four cases of ovarian cancer were diagnosed in their practice each year. There was no difference between the themes for each physician/administrator sampling group, thus the results are presented for providers overall.

**Table 1.** Demographic Characteristics of Provider Participants by Administrator Versus Physician Role

	Physicians, n (%)	Administrators, n (%)
Location of practice		
Urban	5 (83)	3 (75)
Rural	1 (17)	1 (25)
Employment arrangement		
Affiliated with a hospital	3 (50)	4 (100)
Private practice	3 (50)	0 (0)
Area referral practice		
Practice in area with higher number of ovarian surgeries with no gynecologic oncologist^a^	2 (33)	2 (50)
Practice in area with lower number of ovarian surgeries with no gynecologic oncologist^b^	1 (17)	2 (50)
Recently referred patient to hospital with gynecologic oncologists^c^	3 (50)	0 (0)
Years in practice		
<20 years	3 (50)	4 (100)
20+ years	3 (50)	0 (0)

^a^ Five hospitals without a gynecologic oncologist were identified as high-volume ovarian cancer surgery sites through the State Cancer Registry. One administrator, 1 obstetrics-gynecologist, and 1 medical oncologist were sampled and targeted for recruitment from each hospital.

^b^ Five hospitals without a gynecologic oncologist were identified as low-volume ovarian cancer surgery sites through the State Cancer Registry that matched the high-volume hospitals in residency status, bed size, and location in the state. One administrator, 1 obstetrics-gynecologist, and 1 medical oncologist were sampled and targeted for recruitment from each hospital.

^c^ In the final step of recruitment, obstetric-gynecologists and medical oncologists were recruited in sets of 10 until saturation was achieved based on their recent referral of a patient to the only National Cancer Institute hospital in the state affiliated with gynecologic oncologists. The providers had both provided cancer-directed treatments before the referral and had not provided cancer-directed treatments before the referral.

Of the 40 eligible patients with ovarian cancer who were sent recruitment letters, 16 participated in interviews. The current cancer status of interviewees varied from cancer-free to in remission to progression with estimated survival <6 months. Most patients drove 5–25 miles to receive their surgical care. Most patients reported that they traveled to, and attended, appointments with a family member, most often their husband. Two women had emergent surgery where the Emergency Department admitted them to the hospital for surgical care within 24 hours of symptom recognition. Most patients were older than 65 years and had early stage disease (Table 2).

**Table 2.** Demographic Characteristics of Patient Participants

	Patients, n (%)
Age	
<65	6 (38)
65+	10 (62)
Hospital bed size	
1–99	5 (31)
100–299	8 (50)
300–399	3 (19)
Hospital type	
Government, nonfederal	3 (19)
Non-government, non-for-profit	13 (81)
Insurance at time of diagnosis	
Private	4 (25)
Medicare	10 (63)
Unknown	2 (12)
Location of residence	
Rural	7 (44)
Urban	9 (56)
Stage at diagnosis	
I–II	11 (69)
III–IV	5 (31)

Themes that emerged from each group are described later. Illustrative quotes from providers for each theme are presented in Table 3, and quotes from patients are presented in Table 4.

**Table 3.** Illustrative Quotations from Providers

Providers	
System-level barriers to receiving surgical care by a gynecologic oncologist	
Poor provider-to-provider communication	“Like my nurse yesterday spent an hour and 14 minutes. I was sitting right next to her, so I know she spent that much time, trying to get a patient referred, where I'd already talked to [the gyn onc]. She said it was fine. My nurse got transferred, I think, literally eight times. An hour and 14 minutes is just such a waste of my nurse's time. With as busy practice as I have, it has to be really efficient. Finally she talked to someone that was really helpful, but it took her way, way, way too long to do that.”
	“Well, I can think of a particular challenge right now this minute is I want to talk to somebody down there and I know the process for getting in touch with an obstetrician, but if you have questions about a person, it seems like it's not quite as easy to talk about referral … or I mean just to ask them questions about what their opinion is.”
Long time-to-surgery wait times	“But I think if you don't understand how the medical system works, it seems like it sometimes takes too long….I think that's how people see it.….”
	“I think sometimes the concern is maybe the length of time that the patient has to wait to get those appointments.”
Limited number of providers working in referral range	“In general for women's health, I think we're at a crux where there's just not enough providers.”
	“Lack of physicians—I want to see, because we've been blessed to have one here in Des Moines, one to two, depending on, you know, the kind of situation; I don't think there's anything in Des Moines now with the skills necessary to do GYN oncology. So when this guy retires, there's going to be nobody.”
Patient-level barriers to receiving surgical care by a gynecologic oncologist	
Intimidation by tertiary care centers	“So that's a problem. Then my patient feedback is just that it's challenging to go to a big university setting. Lots of people are rounding on the patient. There's lots of people. It's hard to find it, which elevator to take. Just the functionality of it is difficult.”
	“They just think that the university is such a big place and so they feel like they're going to get lost or it's just overwhelming for them to go out there.”
Cost of care at tertiary care centers	“That's what it boils down to is, patients either need a prior auth’ or they're not sure their insurance will cover the referral to Iowa City.”
	“Then they've usually have already checked with their insurance about referrals and if it's in network, or out of network.”
Long travel times to tertiary care centers	“First off, it's the drive. Unfortunately, a lot of the individuals that we are diagnosing with some of these cancers are older and so the drive can be a little bit of a challenge, especially in the wintertime for some of these people.”
	“They have a hard time getting transportation. It's very difficult to get them to go to Iowa City or to Rochester certainly.”
Dissatisfaction leaving their home institution	“I think it's more a lack of familiarity…..And I think there's still just a lot of people, especially in the rural areas around Des Moines, that don't understand why they would have to leave tomorrow. They think that they should be able to get everything here.”
	“There were just some people that refused to leave.”
Perception of decision-making roles	
Patients trust physicians	“They have a longstanding, good relationship with their patients. So, patients for the most part are fairly willing to go if we tell them, that's the best place for them.”
	“In primary care I would have said, very influential or extremely.”
Patient refusal is not an issue	“I think I must be fairly, what do you call it, charismatic, or I don't know, somehow convincing. I don't know which one you want to call it, but I almost never [have a patient refuse].… I just phrase it in a way that makes it relatable, like, ‘Hey, this is what I would do for my own family members, so I think that I'm providing you with the care that I think is the very, very best. While I'd love to operate, that doesn't necessarily mean that's the right thing for me to do here.’”
	“I don't really have a hard time influencing them, I guess.”
Patient requests are rare	“I would say it's pretty infrequent that they request [a specific physician or hospital].”
Reflections on the hypothetical care of a loved one	
Importance of high-quality care	“Just the quality of care. It has to be up to date. It has to be all encompassing. They have to be at least respectful to my patients. They don't have to necessarily be nice, but they've got to be respectful.”
	“I think it's important to send a person to, that has the most cutting edge care and that's what I felt. And it's close proximity to the patients.. And so if we can get just as good of care or maybe even better care at a closer location, I think that's a smart thing to do.”
	“I would tell them that they needed to be taken care of by a board certified, GYN Oncologist.”

**Table 4.** Illustrative Quotations from Patients

Patients	
System-level barriers to receiving surgical care by a gynecologic oncologist	
Patient-level barriers to receiving surgical care by a gynecologic oncologist	
Pain	“The reason I didn't get taken there was because it was an emergency, and I was in intractable pain.”
Shock	“You know, I was so blown away by the diagnosis, and not having had surgery since I was 15 years old… At the point of this surgery, I was 80. I had not had any surgery since I was 15. I guess you would say I was in shock. I wasn't smart enough to ask the surgeon how many times he'd done this, but I guess, in my mind, I thought if my family doctor recommended him, that should be good.”
	“I had just had the worst news of my life and again, completely overwhelmed. I think shellshocked. Incapable of making decisions. It was all rolling over you like a big boulder.”
Perception of decision-making roles	
Trust in physicians	“I think I totally had a choice, they didn't say, ‘We have to do this.’ But they also didn't say, ‘Maybe you should get a second opinion.’ I just trusted them from the start. Maybe if they had said, ‘If you want to get another opinion we won't be bothered by it,’ but they didn't say that and I trusted them so I stayed with him.”
	“_______ is our clinic, and that's where all our doctors are. We just go there…. just the clinic doctor, the woman's doctor. And he decided the surgeon. We don't question. They're good doctors. We don't question what they say is okay. There are good doctors over there.”
Reluctance to seek second opinions	“For better or worse. I did pretty much what I was told to do.”
	“I always feel bad if someone says, ‘Get a second opinion,’ it makes your own doctor feel like you don't trust him.”
Assumptions about surgeons' experience level	“I just know he's considered a very outstanding doctor in lots of different hospitals.”
	“I don't know that I asked that [experience level], but I had a sense of confidence just based on the way that she was talking.”
Minimal online research	“Yes. I did. I did look up internet, but I got a lot of information from my gynecologist.”
	“I probably did at least look up the [hospital], treatment of it [ovarian cancer], and so forth. But other than that, I don't think I did.”
Reflections on the hypothetical care of a loved one	
Willingness to refer their family to the same provider	“Well, since it all came out all right, I guess I probably should recommend him.”
	“[yes], she was very caring, answered all my questions.”

### System-level barriers to receiving surgical care by a gynecologic oncologist

#### Providers

Although providers stated that they thought the health care system had referral options that provided excellent, up-to-date care to patients with ovarian cancer, they reported that their referrals were limited by poor provider-to-provider communication, long time-to-surgery wait times, and the limited number of gynecologic oncologists in their referral range. With regard to poor communication, providers described having difficulty contacting gynecologic oncologists because they were often connected to a resident or fellow when they desired to speak with an attending gynecologic oncologist and scheduling an appointment or transferring records took much time and effort. In addition, they reported that follow-up communication was inadequate or non-existent after the referral was made and surgery was conducted.

On the other hand, providers with personal connections to a gynecologic oncologist, often related to having trained together in a residency program or having a long referral history together, were able to contact the gynecologic oncologists directly and bypass the common scheduling/follow-up barriers. Providers thought long wait times of up to and >3 weeks were stressful for patients and created the perception of inadequate options available in the health care system. Finally, a salient point discussed by providers was the limited number of gynecologic oncologists to whom their patients could be referred.

#### Patients

Patients denied facing any health care system-level barriers. When they described their treatment courses, referrals, physician recommendations, treatment options, and second opinions, referral to a gynecologic oncologist was not mentioned, nor was the awareness of the importance of a referral to a gynecologic oncologist. It, therefore, appeared that an unrecognized barrier that the patients faced was not being referred to a gynecologic oncologist for their initial surgery.

### Patient-level barriers to receiving surgical care by a gynecologic oncologist

#### Providers

A salient theme among providers was that they perceived that patients faced many barriers obtaining surgical care by a gynecologic oncologist, including fear, expense, time, and different goals of care. Providers believed that their patients were intimidated by the thought of receiving care at a large, tertiary referral center. The providers particularly emphasized that patients were confused by the intricacies of parking, worried about navigating the many elevators and hospital wings, and were overwhelmed by the number of providers (*e.g.*, residents, fellows, and medical students) involved in their daily care.

Providers also expressed that care at a tertiary care center led to greater costs for patients, because they were asked to pay for parking, lodging, and, most importantly, out-of-network insurance charges. The long drive time to tertiary care settings was also perceived by providers to be a barrier for patients. Driving longer distances and spending more time in the car was reported to be most concerning when patients were older and receiving care in the wintertime. Finally, providers reported believing that patients generally had a strong preference for local care. They suggested that the commonly held patient goal of receiving care “at home” was a barrier that would be difficult to overcome.

#### Patients

Patients broadly denied facing any personal barriers, and specifically denied the provider-perceived barriers that were read to them as probing questions. Patients purported they could have traveled and been treated anywhere. They reported that they had many choices available to them and expressed a willingness to go to wherever their doctor recommended.

The two patients who had emergency surgery noted that they should have tried to drive to a bigger hospital with a better surgical center but did not think they could make the longer drive due to their pain level. A salient personal barrier faced by non-emergent patients, regardless of stage at diagnosis, was the shock they felt receiving their initial diagnosis. The shock was often described as worse at diagnosis but with lingering effects months later. Many said the shock reduced their ability to make decisions and investigate their options and increased their need to rely on others for help with decision making.

### Perception of decision-making roles

#### Providers

A common theme among providers was that they were very influential in the surgeon selection decision-making process. Providers perceived that patients trusted them and almost always received care at the referral location they recommended. Providers did not believe there were issues with patients refusing to go where they were referred. Most stated that even when patients were hesitant, they could convince the patient to obtain care where they recommended with the explanation that it was the best decision for their health and survival. Providers discussed the importance of considering patient referral requests, but they indicated that patients infrequently had a request or preference for a specific tertiary referral location.

#### Patients

A prominent theme among patients was that they trusted their providers to give the best care possible, whether that involved surgical care or providing an appropriate referral. Patient confidence in providers was primarily based on their role as the provider, their bedside manner, their ability to effectively explain the care plan, and endorsement from a loved one or another trusted provider. Patients generally did not seek nor receive second opinions, because they trusted their providers and thought it would be disrespectful to their provider.

Patients also did not inquire about or assess their surgeon's experiences or qualifications before surgery due to trusting their provider and believing that it was not their place to question the provider. A few patients did some research online about their treatment options but categorized their research as minimal.

### Reflections on the hypothetical care of a loved one

#### Providers

When providers were asked how they would advise a loved one diagnosed with ovarian cancer, they largely recommended receiving surgical care from a gynecologic oncologist at a tertiary care center. The specific hospital system they chose was based on the quality of care available, the up-to-date practices and treatments, availability of cutting-edge research and care, the respectfulness of physicians, the ability to care for comorbid conditions, and proximity to the loved one.

#### Patients

Fourteen out of 16 patients said they would recommend their loved one receive ovarian cancer surgical care from the non-gynecologic oncologist surgeon who performed their primary surgery. Of the two patients who would not have referred a loved one to their previous surgeon, one had recurrence of cancer and was told by a subsequent gynecologic oncologist that the first surgeon had not removed a large portion of the mass, and the other was given a permanent ileostomy and believed that another surgeon would have told her she had other options.

Patient recommendations for their loved ones aligned with their overall satisfaction with the surgeon and willingness to choose the same surgeon if they could go back in time. The latter appeared to be based largely on their surgical outcomes and the surgeon's personality and bedside manner.

## Discussion

Our findings reflect that providers perceived many system-level and patient-level barriers that can impact receipt of surgical care by a gynecologic oncologist. In contrast, patients who had not received surgical care from a gynecologic oncologist perceived no system-level barriers and denied having any of the patient barriers postulated by providers.

Patients did appear to have two important barriers that were not identified by the providers interviewed in our study: (1) They were not referred to a gynecologic oncologist, and (2) the shock of their cancer diagnosis limited their ability to make well-informed surgical care decisions; they were essentially reliant on the diagnosing physician's referral. Both providers and patients converged in their decision-making perceptions that providers were very influential, and patients trusted their providers' recommendations.

Providers' recommendations for a loved one with ovarian cancer suggested that physicians prioritize specialty training, followed by availability of up-to-date care, and proximity of hospital systems with a gynecologic oncologist. With one exception, patients appeared to remain unaware of the importance of a gynecologic oncologist and prioritized a provider with a history of good outcomes and a personable, communicative bedside manner.

There was a divergence in provider perceptions of patient barriers and patient-identified barriers. Provider-perceived distance of care was a barrier and was a factor they considered when advising a loved one about ovarian cancer. This differs from prior ovarian cancer studies and from patient perceptions in this study, but it aligns with findings for other types of cancer.^23,36–39^ Although insurance is commonly found to be a determinant of cancer care, it was not a driver of patient decision making.^40^ In fact, many patients stated they could have received care anywhere in the country, naming large private hospitals as examples.

It is unclear why there is a disconnect between provider-perceived barriers and patient-perceived barriers. It is possible that provider perceptions about patient barriers stem from other diseases or conditions with better prognoses relative to ovarian cancer, such as benign gynecology or other gynecologic cancers, and are then attributed incorrectly to patients with ovarian cancer. Providers should be mindful to avoid generalizing norms to the small number of patients they diagnose with ovarian cancer each year, because these patients appear to recognize the seriousness of ovarian cancer, the most deadly gynecologic cancer, and are largely willing and able to receive care wherever their providers recommend.

Our study yielded results that differed from previously hypothesized explanations of reasons that women with ovarian cancer were not referred to gynecologic oncologists. Unlike previous studies hypothesizing that non-gynecologic oncologists may not have known their patients had ovarian cancer when they initiated surgery, or that there may be a lack of consensus among physicians that surgery for ovarian cancer should be performed by gynecologic oncologists,^21,22^ none of the women interviewed in our study reported having their cancer discovered during surgery, and physicians adamantly stated that all patients with ovarian cancer should be seen by a gynecologic oncologist.

Different from previous studies suggesting that rurality and distance were barriers to care from gynecologic oncologists,^11^ no women in our study, including those residing in rural areas, endorsed these as barriers. Finally, unlike studies citing health insurance as a potential barrier,^3,7,22,23^ most women indicated that insurance was not an issue and they could have gone to any tertiary care hospital.

Larger population-based studies are needed to determine whether diagnosing providers are failing to provide referrals to gynecologic oncologists because they erroneously assume patients cannot travel to larger hospitals where gynecologic oncologists typically practice, or if there are other barriers that were not captured in our study. This study suggests that patients may be limited by provider referrals.

Shared decision making can be complex, especially when care decisions surround cancer surgery.^41^ Patients in our study trusted their providers and did what they recommended. Further, the patients discussed the limitation of the shock of diagnosis and needing to rely on others for help making their decisions. This is consistent with prior findings about shared decision making during ovarian cancer diagnosis.^41^ These findings reinforce the importance of primary care providers, obstetric-gynecologists, oncologists, general surgeons, and emergency medicine physicians strongly referring patients to gynecologic oncologists for surgical care. Patients may find relief in understanding a recommendation exists and limiting the ambiguity of their decision-making process.

### Limitations

Our exploratory findings from a small sample in a single Midwestern state may not be generalizable to all regions, particularly since only White women participated. The goal of our qualitative approach was to elicit in-depth, detailed information about the experiences and attitudes of the patients and providers in our sample rather than to generalize to other populations. Of note, patients were recruited from a population-based cancer registry and providers were recruited from a variety of practice settings.

Selection bias may have resulted due to a low provider participation rate. Patient responses could have been subject to recall bias and provider responses could have been subject to reporting bias, particularly social desirability bias if they believed their answers needed to align with NCCN guidelines when speaking with ICR staff. To reduce the risk of these biases, we employed strategies such as interviewer training, probes, and the option to skip any questions. To be included in our study, patients with ovarian cancer had to survive 4–9 years after diagnosis. This could have selectively excluded patients diagnosed with advanced stage disease.

## Conclusion

Providers and patients disagree on the barriers to receiving care from a gynecologic oncologist. The results of this study emphasize the need for open discussions between patients with ovarian cancer and their diagnosing providers about options and clinical guidelines for surgical ovarian cancer care. Given that patients are overwhelmed at the time of diagnosis and they trust their providers, receipt of care from gynecologic oncologists largely depends on the diagnosing providers' recommendations.

Further research is needed to develop and evaluate mechanisms to improve provider-to-patient discussions about surgical recommendations. In addition, future studies should investigate strategies to reduce perceived barriers for diagnosing providers when they are making referrals so that all women with ovarian cancer have an equal opportunity of receiving guideline-compliant care with the highest rates of survival.

## Supplementary Material

Supplemental data

Supplemental data

Supplemental data

Supplemental data

## Abbreviations Used

ICR: Iowa Cancer Registry
NCCN: National Cancer Center Network
SEER: Surveillance, Epidemiology, and End Results
