# Supplementary material for: Patient and Provider Perspectives on Barriers to Accessing Gynecologic Oncologists for Ovarian Cancer Surgical Care
Source: Womens Health Rep (New Rochelle). 2020 Dec 28;1(1):574–83. doi: 10.1089/whr.2020.0090 (PMC9380881; doi:10.1089/whr.2020.0090)
Supplement: Supplemental data [file Supp_TableS1.docx]

**Supplemental Table 1. Administrators Interview Guide**

| **Question Read Verbatim During Interviews** | **Associated Probing Questions Provided*** |
| --- | --- |
| What is your position? |  |
| How many years have you had this position? |  |
| How would you describe the ownership of your hospital? | Is it independently owned, part of a system, for-profit, non-profit |
| Are there women’s health and gynecologic service physicians affiliated with your hospital system? | If yes, how are they affiliated with your hospital system? Are they a part of your hospital or external to your hospital? |
| Can your hospital provide comprehensive cancer care to ovarian cancer patients? | Can your hospital provider imaging/staging, pathology, surgery, chemo, radiation? |
| Can you describe what happens once a patient is diagnosed with ovarian cancer by an affiliated provider in your hospital system? |  |
| If an affiliated provider in your hospital system diagnosed a woman with ovarian cancer and wanted to refer her for further care, where would the patient likely get referred? |  |
| What happens if a patient does not want to be referred for treatment or wants to receive all care at your hospital? |  |
| Does your hospital, in your opinion, have an adequate number of referral options for ovarian cancer patients? |  |
| Thinking about your patients and providers, how easy is it for them to schedule an appointment with a referred-to hospital/ health center? |  |
| What barriers does your hospital system face when referring patients for ovarian cancer care? | Patient-related vs. physician-related vs. system-related? |
| On a scale of 1 to 10, how would you rate your satisfaction with your hospital system’s ovarian cancer referral network? | Can you say more about what prompted you to select that rating? |
| How could your hospital system’s ovarian cancer referral network be improved? |  |

* Non-written probing questions were asked by interviewers as needed for clarity. All interviewers knew the goal of the study and had extensive training and experience in conducting qualitative interviewers.
