# Supplementary material for: Patient and Provider Perspectives on Barriers to Accessing Gynecologic Oncologists for Ovarian Cancer Surgical Care
Source: Womens Health Rep (New Rochelle). 2020 Dec 28;1(1):574–83. doi: 10.1089/whr.2020.0090 (PMC9380881; doi:10.1089/whr.2020.0090)
Supplement: Supplemental data [file Supp_TableS4.docx]

**Supplemental Table 4. Patient Interview Guide**

| **Question Read Verbatim During Interviews** | **Associated Probing Questions Provided*** |
| --- | --- |
| Would you describe where you live as urban or rural? ‘Agricultural’ may be another way to describe rural towns. | -About how far did you have to travel to get to your hospital visits (related to the cancer)?  -How did you get to your hospital visits (i.e., you drove yourself, someone else drove you, other—describe).  - Did you have someone to go with you to your doctor appointments for your cancer care? If so how? |
| I’m also interested in understanding about your care path – Do you happen to remember what type of doctor you talked to about your ovarian cancer first? What kinds of doctors did you see thereafter? | - Did you see a primary care or family medicine doctor? You might see this type of doctor for normal check-ups?  -Did you see an obstetrics/gynecologist: this type of doctor treats females, preforming regular exams, pap smears, and delivering babies?  - Did you see a cancer doctor?  - Did you see a doctor that preforms surgery? |
| Thinking back to when you first found out you had ovarian cancer, could you describe how you decided where to go for surgery for your cancer care? |  |
| Who did you turn to for help when you were deciding where to have surgery? Who all did you talk to for advice? | - Whose input did you value the most?  - How much did you feel like you had to go where that person told to you to go?  -What did that person think of your decision on where to receive treatment? |
| Besides the people you mentioned, what other sources of information did you use to learn about ovarian cancer surgical treatment? | - Did you use the internet? Talk to friends or family? |
| Did anything make it hard for you to choose a surgeon or hospital? | - did you discuss the other options prior to surgery or after?  -what other options did you discuss?  - why did you decide not to pursue those other options? |
| Surgeons have different levels of experience in performing surgery to remove ovarian cancer. How would you describe your surgeon’s experience level: a) Extremely experienced, b) Very experienced, c) Moderately experienced, d) Slightly experienced, e) Not at all experienced, f) I don’t know | - Why did you choose that answer?  - Did you discuss their experience with performing surgery for ovarian cancer prior to the surgery? |
| How satisfied are you with the surgeon you chose? a) Extremely satisfied, b) Very Satisfied, c) Moderately Satisfied, d) Slightly satisfied, e) Not at all satisfied | If you had to do everything over again, would you choose the same surgeon?  Would you recommend this surgeon to a friend or family member?  What kinds of things made you satisfied/unsatisfied with the surgeon? |
| How much did you feel like you had a choice of which surgeon or hospital to go to for surgery? |  |
| What barriers did you face in receiving ovarian cancer surgery? | - Were your options limited due to distance to care, insurance, or ability to travel? |
| How would you describe your overall physical health at the present time? |  |
| How would you describe your mental and emotional health at the present time? |  |
| How would you have described your health prior to being diagnosed with cancer? | - Did you have any major health issues before your cancer? |

* Non-written probing questions were asked by interviewers as needed for clarity. All interviewers knew the goal of the study and had extensive training and experience in conducting qualitative interviewers.
