# Supplementary material for: Patient and Provider Perspectives on Barriers to Accessing Gynecologic Oncologists for Ovarian Cancer Surgical Care
Source: Womens Health Rep (New Rochelle). 2020 Dec 28;1(1):574–83. doi: 10.1089/whr.2020.0090 (PMC9380881; doi:10.1089/whr.2020.0090)
Supplement: Supplemental data [file Supp_TableS2.docx]

**Supplemental Table 2. Obstetrics-Gynecologist Interview Guide**

| **Question Read Verbatim During Interviews** | **Associated Probing Questions Provided*** |
| --- | --- |
| What type of residency and/or fellowship training have you completed? |  |
| How many years have you been in practice? |  |
| Are you in a solo vs. group practice, or hospital employed? | If group practice, how many people are in your group?  If solo or group, are you affiliated with a single health system or multiple health systems?  How large is your patient load? |
| How many times per year would you estimate that you diagnose ovarian cancer? |  |
| Thinking back to the last patient you diagnosed with ovarian cancer, can you describe the process you went through to arrange treatment for her? | Is that typical?  If no, describe a typical process you go through to arrange treatment for ovarian cancer patients |
| Do you typically refer patients elsewhere? | How do you decide where to refer patients?  How often do patients request a referral to a specific physician or a hospital where they want to receive treatment?  How satisfied are you with the referral options available to your patients?  What challenges do you experience when referring patients for treatment?  (e.g., Patient-related vs. physician-related vs. system-related?) |
| How often are there times when the patient refuses to go elsewhere for treatment? | What reasons do they discuss?  Do they discuss any of the following as reasons?  Lack of transportation  Financial burden  Lack of trust or familiarity with other hospitals  Other |
| Do you treat any patients yourself? | Do you perform surgery? If so, for what percentage of your ovarian cancer patients do you perform surgery?  Do you manage chemotherapy for ovarian cancer patients? If so, for what percentage of your ovarian cancer patients do you manage their chemotherapy? |
| How would you describe the level of influence you have over where your patients with ovarian cancer ultimately receive care? Would you say you are: a) Extremely influential, b) Very influential, c) Somewhat influential, d) Slightly influential  e) Not at all influential |  |
| Where would you recommend someone close to you go for surgical treatment if they were diagnosed with ovarian cancer? | What factors would most strongly influence your recommendations? |

* Non-written probing questions were asked by interviewers as needed for clarity. All interviewers knew the goal of the study and had extensive training and experience in conducting qualitative interviewers.
